# Loss of parkin reduces lung tumor development by blocking p21 degradation

**DOI:** 10.1371/journal.pone.0217037

**Published:** 2019-05-21

**Authors:** Kyung-Ran Park, Jae Suk Yun, Mi Hee Park, Yu Yeon Jung, In Jun Yeo, Kyung Tak Nam, Hae Deun Kim, Ju Kyoung Song, Dong-Young Choi, Pil-Hoon Park, Sang-Bae Han, Hyung-Mun Yun, Jin Tae Hong

**Affiliations:** 1 Department of Oral and Maxillofacial Pathology, School of Dentistry, Kyung Hee University, Dongdaemun-Gu, Seoul, Republic of Korea; 2 College of Pharmacy and Medical Research Center, Chungbuk National University, Osong-eup, Heungduk-gu, Cheongju, Chungbuk, Republic of Korea; 3 College of Pharmacy, Yeungnam University, Gyeongsan, Gyeongbuk, Republic of Korea; University of South Alabama Mitchell Cancer Institute, UNITED STATES

## Abstract

Several epidemiological studies have demonstrated the reciprocal relationship between the development of cancer and Parkinson’s disease (PD). However, the possible mechanisms underlying this relationship remain unclear. To identify this relationship, we first compared lung tumor growth in parkin knockout (KO) mice and wild-type (WT) mice. Parkin KO mice showed decreased lung tumor growth and increased expression of p21, a cell cycle arrester, as compared with WT mice. We also found that parkin interacts with p21, resulting in its degradation; however, parkin KO, knockdown, as well as mutation (R275W or G430D) reduced the degradation of p21. We investigated whether parkin KO increases the association of p21 with proliferating cell nuclear antigen (PCNA) or CDK2 by reducing p21 degradation, and, thus, arresting the cell cycle. The interaction between p21 and PCNA or CDK2 was also enhanced by parkin knockdown, and this increased interaction induced sub G0/G1 arrest, leading to cell death. Therefore, our data indicate that parkin KO reduces the development of lung tumors via cell cycle arrest by blocking the degradation of p21. These findings suggest that PD could be associated with lower lung cancer incidence.

## Introduction

A recent cohort study of 406 patients with Parkinson's disease (PD) showed lesser cancer incidence in the total number of patients compared to that in normal individuals [1–2]. It has been reported that the incidence of prostate, lung, bladder, stomach, colorectal, leukemia, and uterus cancers is negatively associated with the development of PD, while that of breast, non-melanoma skin, and brain cancers is positively associated with the development of PD [3–4]. Parkin is a RING-between-RING E3 ligase that functions to ubiquitinate specific substrates. PARK2 functions are implicated in a wide variety of biological processes that are involved in the regulation of cell growth and survival, including the cell cycle, mitochondrial homeostasis, metabolism, xenophagy, protein turnover, and stress responses. Mutations in parkin have also been linked to PD [5]. It has been reported that the parkin gene family members (PARK1/4, PARK2, PARK5, PARK6, PARK7, PARK8, PARK9, and PARK15) were overexpressed in the tumors of patients with non-small cell lung cancer (NSCLC) [6].

Lung cancer is one of the most common cancers and the leading cause of cancer deaths worldwide. Approximately 80–85% of lung cancers are NSCLC, while about 10–15% are small cell lung cancer. It has been reported that the expression of p21, a cell cycle regulator, is lower in patients with NSCLC. Furthermore, these patients were found to have a significantly shorter overall survival [7]. It was reported that p21, as an inhibitor of the CDK/cyclin complex, is activated in response to a variety of cellular and environmental signals to suppress tumor growth [8]. Higher tumor susceptibility was also reported in p21 null mice [9]. In addition, the tumor susceptibility of HCT116 p21+/+ human colon cell-bearing nude mice was shown to be less compared to their HCT116 p21–/–counterparts [10]. *Adnane et al*. demonstrated that loss of p21Cip1 enhances the formation of lung tumors in urethane-treated mice, as well as in mammary and salivary tumors of mice expressing the MMTV/v-Ha-ras transgene [11]. *Philipp et al*. also showed that p21Cip1 loss promoted the de-differentiation of squamous carcinomas [12]. Furthermore, Jackson and colleagues demonstrated that p21Cip1-null mice exhibited accelerated tumor onset and increased tumor multiplicity after urethane treatment [13]. These reports indicate that p21 has significant roles in tumor suppression.

It is known that parkin accelerates the degradation of ataxin-2 [14], p38[15], β-catenin [16], programmed cell death-2 [17], dynamin-related protein [18], Bcl-2 [19], cell division cycle related-1 [20], and Hsp70 [21]. Notably, the RING domains on parkin have been shown to recruit ubiquitin conjugating enzymes, such as E2 ligases, thereby facilitating the binding of E3 ligase and leading to substrate degradation [22]. Several studies have reported that p21 acts as a substrate of the E3 ubiquitin ligase. It was reported that E3 ubiquitin ligases, such as SKP1–CUL1-SKP2 (SCF^SKP2^), CUL4A, or CUL4B–DDB1-CDT2 (DDB1 is DNA damage-binding protein 1) (CRL4^CDT2^), and anaphase-promoting complex (APC)-cell division cycle 20 (APC/C^CDC20^) promote the proteolysis of p21 [23–28]. Recently, we reported that parkin KO inhibits neuronal development via regulation of proteasomal degradation of p21 [29].We, thus, hypothesized that mutations in parkin would inhibit the degradation of p21, leading to cell cycle arrest, which ultimately results in tumor growth inhibition. Therefore, we investigated whether parkin deficiency may prevent tumor growth through the inhibition of p21 degradation in patients with PD.

## Materials and methods

### Cell culture

The normal lung cell line LL24 and lung squamous cell line HCC-1588 were obtained from the Korean Cell Line Bank (Seoul, Korea). The human embryonic kidney cell line HEK293 and lung adenocarcinoma cell line A549 were obtained from ATCC. HEK293 cells were selected for higher transfection efficiency. LL24, A549, and HCC-1588 cells were grown in RPMI-1640 medium (Gibco, Life Technologies, Grand Island, NY) with 10% fetal bovine serum, 100 U/ml penicillin, and 100 μg/ml streptomycin at 37°C in a humidified atmosphere of 5% CO2. HEK293 cells were grown in Dulbecco’s modified Eagle’s medium (Gibco, Life Technologies, Grand Island, NY) with 10% fetal bovine serum, 100 U/ml penicillin, and 100 μg/ml streptomycin at 37°C in a humidified atmosphere of 5% CO2. These cell lines were authenticated by monitoring of cell morphology and by contamination inspection.

### Cell viability assay

Cells were plated in 96-well plates and subsequently transfected for 48 h. After treatment, cell growth was evaluated by MTT [3-(4,5-dimethylthiazol-2-yl)-2,5-diphenyltetrazolium Bromide] assay (Sigma-Aldrich, St. Louis, MO) according to the manufacturer’s instructions. Briefly, MTT (5 mg/mL) was added and plates were incubated at 37°C for 2 h before 100 μL dimethyl sulfoxide was added to each well. Finally, the absorbance of each well was measured at a wavelength of 540 nm using a microplate reader.

### Evaluation of apoptotic cell death

TUNEL assay was performed using the DeadEnd^TM^ Fluorometric TUNEL System (Promega, Madison, Wisconsin, USA) for in situ detection of apoptotic cells, according to the manufacturer’s instructions. NSCLC cells (5 × 10^3^ cells/well) were cultured on 8-chamber slides after transfection with Lipofectamine RNAiMAX reagent. The cells were washed with phosphate buffered saline (PBS) and fixed by incubation with 4% paraformaldehyde in PBS for 1 h at room temperature. The membrane was permeabilized by exposure to 0.1% Triton X-100 in PBS for 5 min at room temperature. For DAPI staining, slides were incubated for 15 min at room temperature in the dark with a mounting medium containing DAPI (Vector Laboratories, Inc., Burlingame, CA) for fluorescence. The cells were then observed under a fluorescence microscope (Leica Microsystems AG, Wetzlar, Germany). The total number of cells in a given area was determined by DAPI and TUNEL staining. The apoptotic index was determined as the number of DAPI- and TUNEL-positive cells divided by the total number of cells counted × 100.

### Human samples

The human breast cancer tissues, liver cancer tissues, colon cancer tissues, lung cancer tissues, neuroblastoma, ovarian cancer tissues, and stomach cancer tissues samples were purchased from US Biomax, Inc. cancer tissue bank collection (US Biomax, Inc., MD, USA).

### Parkin knockout (KO) transgenic mice

Parkin KO transgenic mice were purchased from the Jackson Laboratory, and C57BL/6 mice were purchased from Orient Bio. The mice were housed and bred under specific pathogen-free conditions at the Laboratory Animal Research Center of Chungbuk National University, Korea. Ten mice per cage were maintained in a room with a constant mean 6 SD temperature of 22 ± 18°C, mean 6 SD relative humidity of 55 ± 10%, and a 12 h light/dark cycle. Mice were fed standard rodent chow (Samyang) and purified tap water ad libitum.

### Animal experiments

Eighteen- to twenty-week-old mice were used. Tumors were induced by a single intraperitoneal injection of 1 mg/g urethane (ethyl carbamate; Sigma-Aldrich, St Louis, MO, USA) once a week for 10 weeks. Mice were euthanized at time points up to 6 months after injection of carcinogen. At the time of sacrifice, lungs were lavaged, perfused, and fixed in 4% paraformaldehyde. After fixation, lungs were used for surface tumor number and diameter measurements and embedded in paraffin. Tumors on the lung surface were enumerated by at least two experienced readers and blinded to sample identifiers under a dissecting microscope. Tumor counts were averaged and statistically analyzed.

### Ethics statement

Mice used in this study were maintained in accordance with the National Institute of Toxicological Research of the Korea Food and Drug Administration guidelines for the humane care and use of laboratory animals. All experiments were approved and carried out according to the Guide for the Care and Use of Animals [Animal Care Committee of Chungbuk National University, Korea (approval number: CBNUA-436-12-02). Diethyl ether was used in animal experiments as a euthanasia agent to minimize animal suffering and distress. After appropriate quantity of ether was poured onto cotton wool and allowed to evaporate and fill the chamber, the animals were placed on the ether soaked cotton wool under a mesh so that the animals do not have direct contact with the liquid chemical (ether). After euthanasia, the animal carcasses and the soaked cotton wool were removed and placed inside the chemical fume hood to allow dissipation of the chemical. The extraction system of the fume hood remained switched on for a further period of 30 minutes after the animal carcasses were handled following standard clinical waste procedures.

### Western blotting

Western blot analysis was performed as described previously [30]. The membranes were incubated with the following antibodies: parkin, p21, CDK2, CDK4, CDK6, cyclin D, cyclin E1, and β-actin. (1:1,000 dilution; Santa Cruz Biotechnology, CA, USA), caspase-3, and proliferating cell nuclear antigen (PCNA) (1:1000 dilution; Cell Signaling, Beverly, MA). The blots were treated with specific antibodies followed by incubation with secondary antibodies, and visualization was performed using an enhanced chemiluminescence (ECL) detection system.

### siRNA transfection

A549, HCC-95, and NCI-H1299 NSCLC cells (5 × 10^3^ cells/well) were plated onto 96-well plates and transiently transfected with parkin siRNA for 48 h using a mixture of siRNA and the Lipofectamine RNAiMAX reagent in Opti-MEN, according to the manufacturer’s specifications (Invitrogen, MA, USA). The transfected cells were used for detecting cell viability and protein expression.

### Cell cycle analysis

To examine the cell cycle distribution of asynchronous populations of lung cancer cells, replicative DNA synthesis and DNA content were analyzed using bivariate flow cytometric analysis. Parkin siRNA- or mutant parkin (R275W or G430D)-transfected A549 cells were harvested by trypsin-EDTA release and fixed in ice-cold 70% ethanol. At least 1 to 2 h before flow cytometric analysis, cells were resuspended in a 1 mL aliquot of modified Vindelov's DNA staining solution (10 μg/mL RNase A and 5 μg/mL propidium iodide in PBS). Flow cytometric analysis was performed with the flow cytometry system (FACS Calibur-S System; BD Biosciences, San Jose, CA). Cells in the G1, S, and G2-M phases of the cell cycle were determined with Modfit LT (Verity House Software, Top-sham, ME).

### Immunofluorescence staining

NSCLC cells were plated in the chamber slides at a density of 5 × 10^4^ cells/well cells per chamber. The cells were transfected with parkin siRNA for 48 h. Then, the cells were washed once with PBS, fixed with 4% paraformaldehyde for 20 min, membrane-permeabilized by exposure to 0.1% TritonX-100 for 2 min in PBS and placed in blocking serum (5% bovine serum albumin in PBS) at room temperature for 2 h. The cells were then incubated with primary rabbit polyclonal antibodies against parkin and p21 (1:100 dilution, Santa Cruz Biotechnology, CA, USA) overnight at 4°C. After washes with ice-cold PBS, followed by treatment with an anti-rabbit secondary antibody labeled with Alexa Fluor 488 and 568 (1:100 dilution, Molecular Probes Inc., Eugene, OR) for 2 h at room temperature, immunofluorescence images were acquired using a confocal laser scanning microscope (TCS SP2; Leica Microsystems AG, Wetzlar, Germany).

### Immunoprecipitation

Immunoprecipitation assay was performed as described previously. The cells were lysed in EBC buffer. The precleared soluble supernatants were mixed with a polyclonal anti-p21 antibody and incubated overnight at 4°C. Protein A/G beads were then added to the reaction mixture. After washing the immune complexes, the bound proteins were resuspended in sodium dodecyl sulfate sample buffer, resolved by sodium dodecyl sulfate-polyacrylamide gel electrophoresis, incubated with a monoclonal antibody against parkin, and analyzed by western blotting.

### Octet analysis

Binding interactions were analyzed using an Octet system (Fortebio, Inc., Menlo Park, CA) by measuring the wavelength shift in nanometers. Kinetic rate constants were determined with HIS1K-Anti-penta-His sensor in the advanced kinetic mode. The recombinant His-tagged parkin protein was immobilized on the sensor at 2 μg/mL in 10× kinetic buffer (Fortebio, Inc., Menlo Park, CA) and exposed for 1200 s to various concentrations of recombinant p21 protein covering at the expected value of the dissociation constant (*K*_D_), followed by a 4000 s dissociation step in 10× kinetic buffer with 1000 rpm shaking. Interferometry data were globally fitted to a 1:1 binding ratio for calculating kinetic parameters using the Octet ForteBio software (Fortebio, Inc., Menlo Park, CA).

### In vitro and in vivo ubiquitination assay

In vitro ubiquitination assay was performed according to the manufacturer’s manual (Boston Biochem). HEK293 and A549 cells were transfected with HA-ubiquitin (2 μg), Flag-p21 (1 μg), Myc-Parkin (1 μg) expression plasmids using Lipofectamine 3000. Forty-eight hours after transfection, cells were harvested and split into two aliquots, one for immunoblotting and the other for ubiquitination assay. The eluted proteins were analyzed by immunoblotting with monoclonal p21 antibodies.

### Immunofluorescence staining

The membrane was permeabilized by exposure to 0.1% Triton X-100 for 2 min in PBS and placed in blocking serum (5% bovine serum albumin in PBS) at room temperature for 2 h. The cells were then incubated with a primary rabbit polyclonal antibody against active p53 (1:100 dilution, Santa Cruz Biotechnology, CA, USA) overnight at 4°C. After washes with ice-cold PBS, followed by treatment with an anti-rabbit secondary antibody labeled with Alexa Fluor 488 (1:100 dilution, Molecular Probes Inc., Eugene, OR) for 2 h at room temperature, immunofluorescence images were acquired using a confocal laser scanning microscope (TCS SP2; Leica Microsystems AG, Wetzlar, Germany) equipped with a lens for 200X magnification.

### Immunohistochemistry

Immunohistochemical assay was performed using the avidin-biotin–peroxidase method as described previously [30]. Sections were stained with hematoxylin and eosin (Sigma-Aldrich), anti-COX-2 antibody (Novus), and anti-iNOS antibody (Novus).

### Statistical analysis

The data were analyzed using the GraphPad Prism 4 ver. 4.03 software (Graph-Pad Software, La Jolla, CA). Data are presented as mean ± standard deviation (SD). The differences in data between all groups were assessed by one-way analysis of variance (ANOVA). When the P value in the ANOVA test indicated statistical significance, differences were assessed by the Dunnett’s test. A P value ≤ 0.05 was considered to be statistically significant.

## Results

### Suppressive effects of urethane on lung tumor development in parkin KO mice

**Fig 1:**
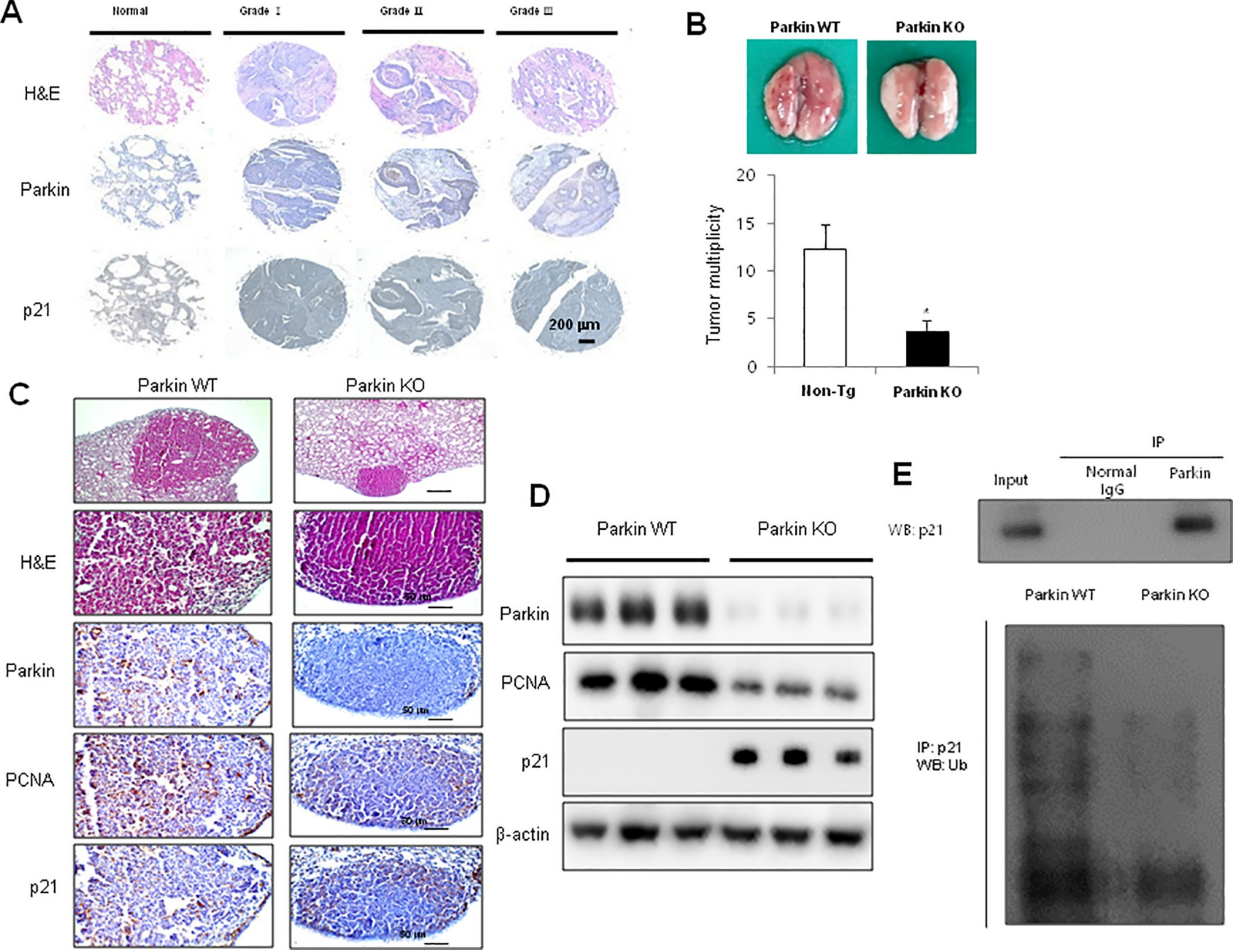
Suppressive effects of parkin KO on urethane-induced lung tumor development. **(A)** Human normal lung or NSCLC tissue sections (Grade I–III) were processed and stained with H&E, and immunohistochemical analysis for the expression of parkin and p21 was performed. The figures represent 3 samples of each cancer stage. **(B)** Effect of IL-32γ on urethane-induced lung tumorigenesis in transgenic mice. The results are expressed as mean ± SD. * P < 0.05 compared with WT mice (n = 10). **(C)** Lung tumor tissue sections were analyzed by immunohistochemistry for the detection of parkin, PCNA, and p21 expression in the tumor tissues of parkin KO and WT mice. **(D)** The expression of parkin, p21, and PCNA was detected in the lung tumor tissues using specific antibodies. β-actin protein was used as an internal control. **(E)** Binding of parkin and p21 was detected using an immunoprecipitation assay (upper lane), while endogenous ubiquitination of p21 was detected through a ubiquitination assay (lower lane) in WT or parkin KO mice. Each band is representative of three independent experiments.

We first examined the expression of p21 and parkin in patients with lung tumor. We observed downregulation of p21 and overexpression of parkin with the progression of lung cancer (Fig 1A). To identify tumor growth effects upon parkin KO, we measured lung tumor growth in a urethane-induced carcinogenesis model. Results revealed that the incidence of lung tumors in urethane-induced parkin KO mice was much lower compared to that in wild-type (WT) mice (Fig 1B). Immunohistochemistry (Fig 1C) and western blot analysis (Fig 1D) showed that the expression of p21 was increased, while tPCNA which is considered to be a marker of cell proliferation in various cancers and parkin were decreased in the lung tumor tissues of parkin KO mice. We also determined endogenous ubiquitination in parkin KO lung tumor tissues and showed that parkin directly binds to p21, and p21 ubiquitination and degradation were decreased in parkin KO mice-derived lung tumor tissues compared to those in WT mice-derived tissues (Fig 1E).

### Interaction between p21 and parkin, and degradation of p21

**Fig 2:**
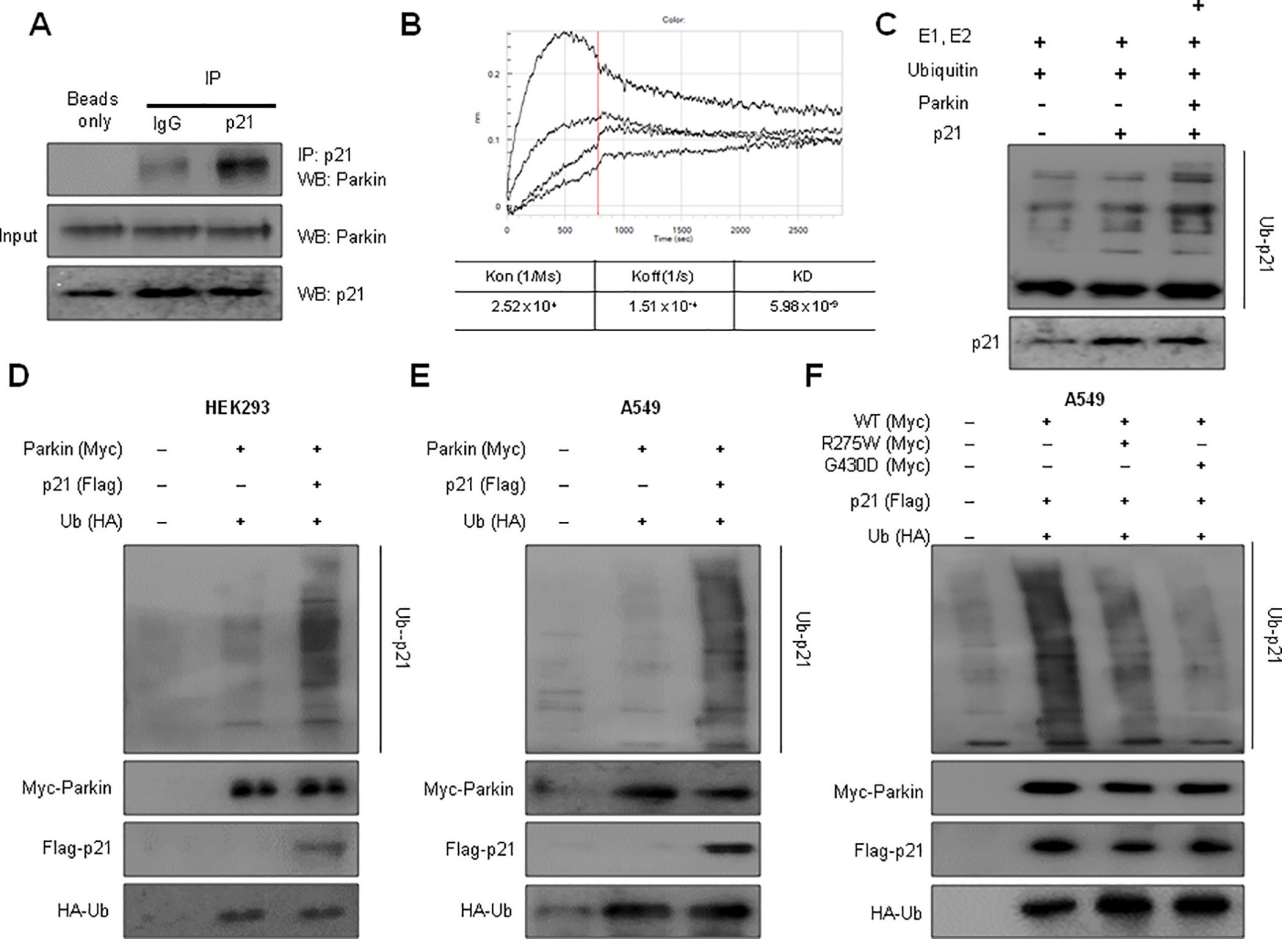
Interactions between p21 and parkin, and the degradation of p21. **(A)** A549 cell lysates were immunoprecipitated with anti-p21 antibody; the cell lysates and immunoprecipitants were then analyzed by immunoblotting with anti-parkin antibody. Interaction between p21 and parkin. **(B)** Representative octet binding kinetic traces from 2 separate experiments with similar results. Full kinetic dataset for the binding ability of p21 to parkin. **(C)** Parkin-mediated ubiquitination on p21 in in vitro and in vivo studies. In vitro ubiquitination reaction of p21 in the presence of parkin. The reaction mixtures were immunoblotted using the indicated antibodies. **(D—E)** In vivo ubiquitination assay of HEK293 (D) and A549 cells (E) transfected with plasmids expressing Flag-tagged p21, Myc-tagged parkin, and HA-tagged ubiquitin. **(F)** In vivo ubiquitination assay of A549 cells transfected with plasmids expressing Flag-tagged p21, HA-tagged ubiquitin, and Myc-tagged WT or G430D/R275W mutant parkin. Cells were treated with MG132 (10 μM) for 6 h and harvested. Ubiquitinated p21 was visualized by western blot analysis using anti-ubiquitin antibody. Each band is representative of three independent experiments.

To study the role of p21 in the inhibitory tumor growth effects of parkin KO, we hypothesized that parkin E3 ubiquitin ligase binds and degrades p21 as a direct substrate. Thus, to test this hypothesis, we performed immunoprecipitation assay. The binding of parkin to p21 was detected by immunoblotting using an anti-p21 antibody. The results indicated that parkin interacted with A549 cell lysates containing p21 (Fig 2A). We also detected an interaction between p21 and parkin using the Octet system. We determined the KD values by association and dissociation experiments for the interaction between p21 and parkin and showed that parkin strongly binds with p21 (KD values = 5.98 × 10−9) (Fig 2B). We then tested whether p21 was a potential target of parkin for ubiquitination using in vitro and in vivo ubiquitination assays. We first performed the in vitro ubiquitination assay and showed that p21 underwent ubiquitination in the presence of parkin, while in the absence of a parkin, p21 was not degraded when compared to first lane which is background signals by ubiqutin protein, and E1 and E2 enzymes.(Fig 2C). To determine whether parkin can mediate protein ubiquitination in vivo, vectors expressing Myc-tagged parkin, HA-tagged ubiquitin, and Flag-tagged p21 were transfected into HEK293 (Fig 2D) and A549 cells (Fig 2E). Cell extracts were subjected to immunoblotting with antibodies against Myc, HA, and Flag. Parkin and ubiquitin were expressed in the transfected cells, resulting in a marked increase in p21 protein ubiquitination. To determine the effects of clinically relevant parkin mutations on the p21 steady state levels using a degradation system, we evaluated ubiquitination using two distinct types of genetic missense mutations. R275W in the RING1 domain and G430D in the RING2 domain of parkin were both presumed to result in the loss of parkin function. We thus co-expressed p21 with HA-ubiquitin and either WT parkin or mutant parkin constructs (R275W, G430D) and performed ubiquitin affinity pull-down experiments. Overexpression of WT parkin resulted in an increase of ubiquitinated high-molecular-weight p21 bands; however, co-expression of either of the parkin mutants resulted in a decrease in the levels of high-molecular-weight p21 species compared with WT parkin and first lane which is background signals(Fig 2F).

### Cell cycle arrest by knockdown of parkin or mutant parkin

**Fig 3:**
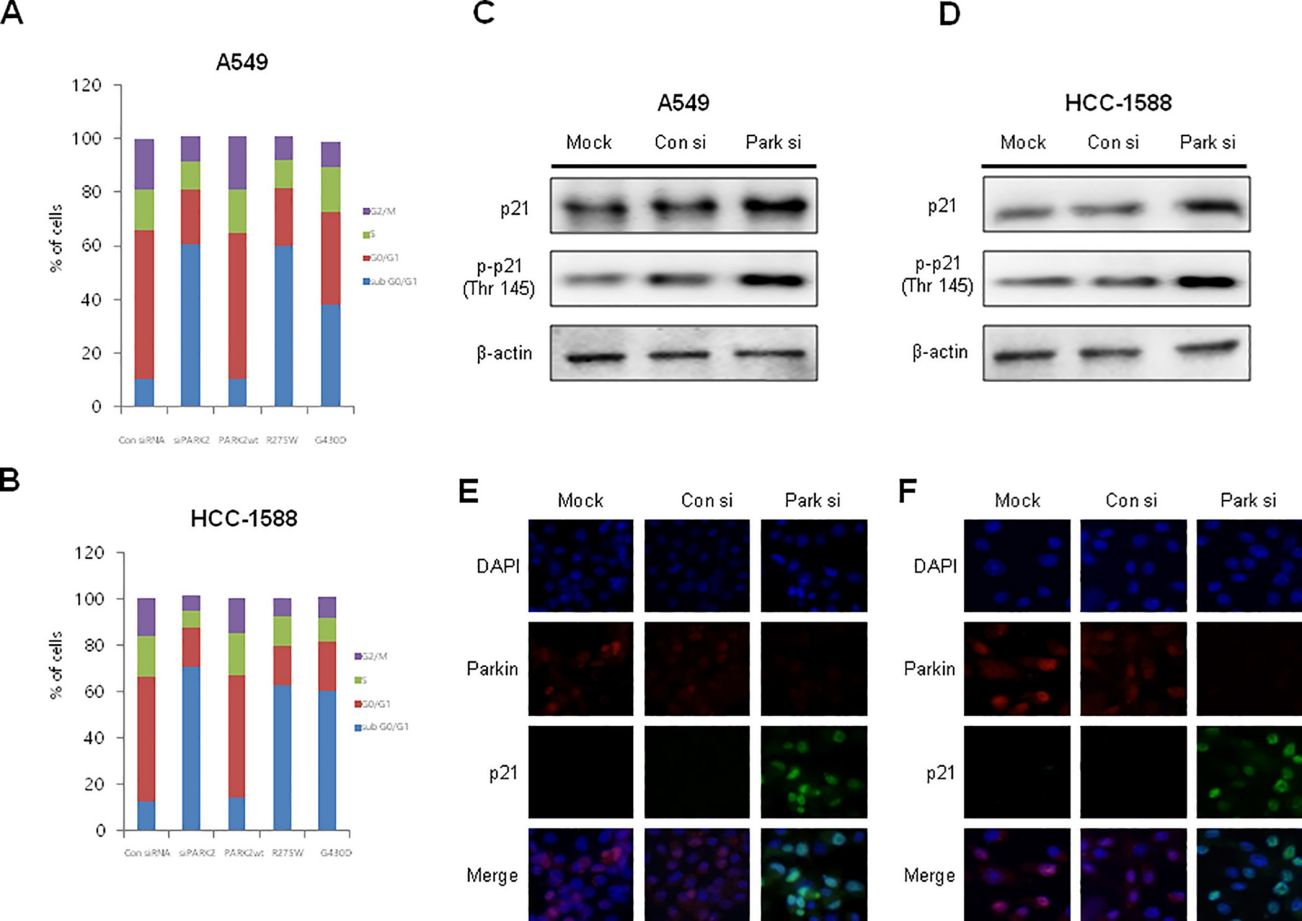
Effects of siRNA or mutant parkins on the cell cycle of NSCLC cells. **(A—B)** A549 cells were transfected with parkin siRNA or mutant parkin (R275W or G430D) for 48 h. The DNA content was analyzed by flow cytometry as described in the Materials and Methods section. Data are representative of three independent experiments performed in triplicates. **(C–D)** Increased p21 expression by parkin knockdown in NSCLC cells. Immunoblotting was used to measure parkin, p21, and phosphorylated p21 expression using specific antibodies. β-actin was used as internal controls. Each band is representative of three independent experiments. (**E**—**F**) We also demonstrated the translocation of p21 by immunofluorescence using a confocal microscope. NSCLC cells were transfected with parkin siRNA for 48 h, and p21 translocation into the nucleus was increased significantly in parkin siRNA-treated NSCLC cells.

In order to determine the effects of parkin knockdown on cell cycle progression, flow cytometry analysis was performed on lung cancer cell lines transfected with parkin siRNA. The results showed that, compared to untransfected control cells, parkin siRNA-treated A549 and HCC-1588 cells were arrested in sub G0/G1 phase (Fig 3A and 3B), and this was accompanied by a significant decrease in the number of cells in the G1 phase. We also observed that mutations in parkin (RING1 domain mutant, R275W and RING2 domain mutant, G430D) caused a sub G0/G1 phase cell cycle arrest in both A549 and HCC1588 cells (Fig 3A and 3B). We then investigated the involvement of p21 in parkin siRNA-induced lung cancer cell cycle arrest, wherein we observed that knockdown of parkin resulted in increased expression of phosphorylation of p21 (Thr 145) (Fig 3C and 3D). We also demonstrated an increase in p21 expression through immunofluorescence, in which p21 was observed to be accumulated in the nucleus of A549 and HCC-1588 cells transfected with parkin siRNA (Fig 3E and 3F).

### Effect of knockdown of parkin on binding between p21 and CDK2 or PCNA in the NSCLC cells

**Fig 4:**
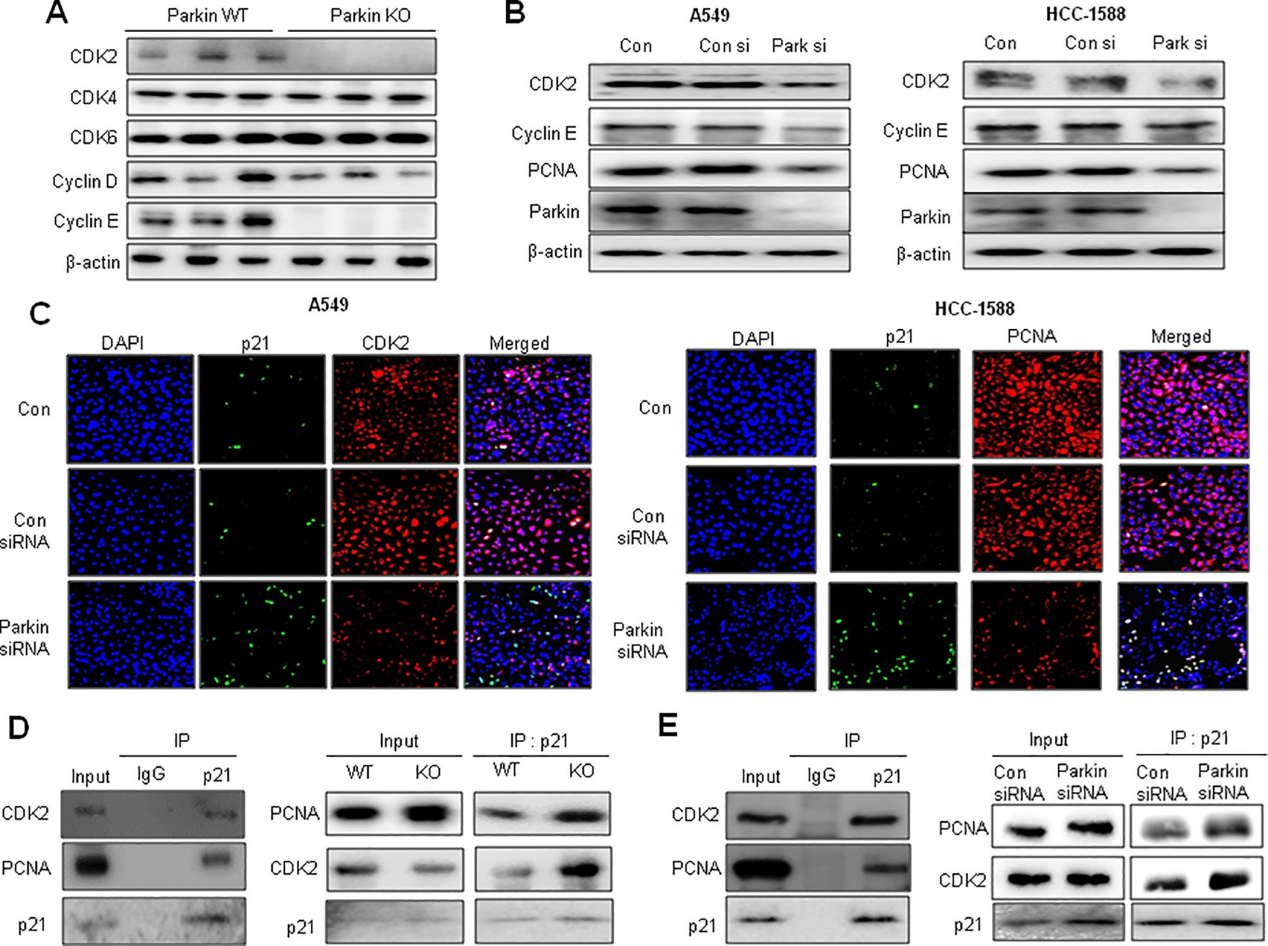
Changes of cell cycle regulatory proteins by loss of parkin. **(A)** Expression of cell cycle regulatory proteins was detected using specific antibodies in the lung tumor tissues of WT or parkin KO mice. β-actin protein was used as an internal control. **(B)** Expression of cell cycle regulatory proteins, including CDK2, cyclin E, PCNA, and β-actin was detected using specific antibodies. **(C)** Co-localization of p21 and CDK2 or PCNA was determined by immunofluorescence using a confocal microscope. NSCLC cells were transfected with parkin siRNA for 48 h. Co-localization of p21 and CDK2 or PCNA increased significantly in parkin KD NSCLC cells. **(D)** Effects of parkin KO on PCNA and the CDK-cyclin complex in lung tumor tissues. Tissue lysates from WT or parkin KO mice were immunoprecipitated with anti-p21, after which the immunoprecipitants were analyzed by immunoblotting with anti-PCNA or anti-CDK2 antibody. **(E)** Lysates of A549 cells transfected with p21 plasmid vectors were immunoprecipitated with anti-p21 antibody. The cell lysates and immunoprecipitants were analyzed by immunoblotting with anti-PCNA and anti-CDK2 antibodies. Lysates of A549 cells transfected with parkin siRNA were immunoprecipitated with anti-p21 antibody. The cell lysates and immunoprecipitants were analyzed by immunoblotting with anti-PCNA and anti-CDK2 antibodies.

Generally, p21 protein binds to CDK2 or PCNA and inhibits their function as cell cycle progression regulators [28, 31]. Thus, we investigated the expression of PCNA and other cell cycle regulatory proteins in lung tumor tissues and lung cancer cell lines by immunoblotting after treatment with parkin siRNA. Among several cell cycle regulatory proteins, the expression of CDK2 and PCNA was dramatically decreased in the lung tumor tissues of parkin KO mice compared with that in WT mice (Figs 1D and 4A) and parkin siRNA-transfected lung cancer cells (Fig 4B). In contrast, knockdown of p21 resulted in increased expression of CDK2, cyclin E, as well as parkin, accompanied with increased cell growth in both A549 and HCC-1588 NSCLC cells (S1 Fig). We also observed a decrease in both CDK2 and PCNA expression, but the opposite effect was observed for p21 expression in parkin siRNA-transfected A549 and HCC-1588 cells by immunofluorescence analysis (Fig 4C). We then investigated whether increased p21 expression by parkin siRNA led to a gain-of-function in p21 by enhancing the association between p21/CDK2 or p21/PCNA complexes. We showed that p21 interacts with CDK2 or PCNA, and their interaction was increased in parkin KO mice compared with that in WT mice (Fig 4D). We also observed that p21 directly interacts with CDK2 or PCNA, and the interaction between p21/CDK2 or p21/PCNA following parkin siRNA treatment was increased in A549 lung cancer cell lines (Fig 4E).

### Effects of parkin siRNA on cell growth and apoptotic cell death in NSCLC cells

**Fig 5:**
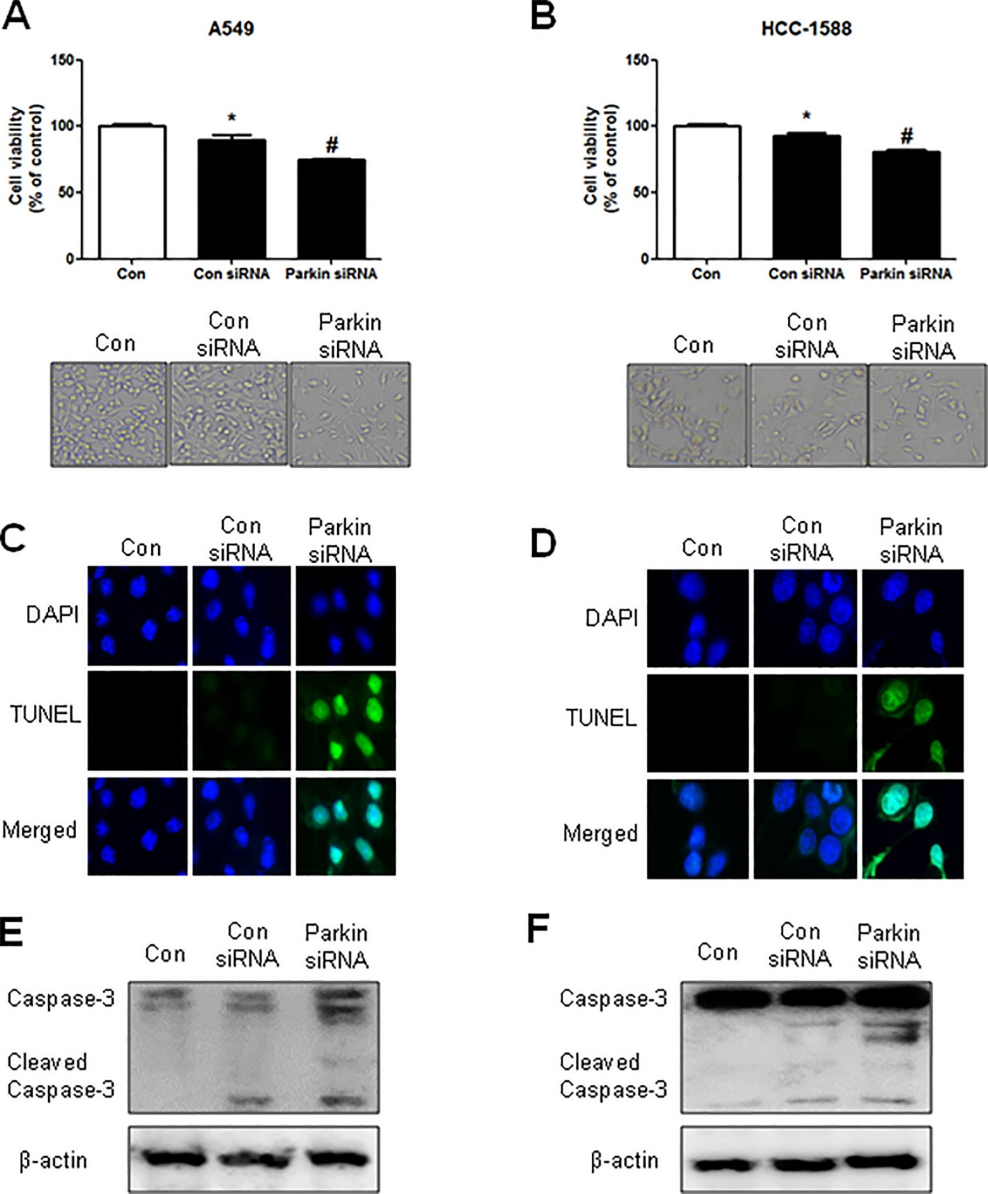
Effects of parkin knockdown on NSCLC cell growth and apoptotic cell death. **(A—B)** Human NSCLC cells were transfected with parkin siRNA for 48 h. After transfection, cell viability was measured by the MTT assay. The morphological changes in A549, HCC-1588, and NCI-H1299 cells were observed by phase contrast microscopy (lower panel). The data are expressed as the mean ± SD of three experiments. *P ≤ 0.05 indicates statistically significant differences from the control group. **(C—D)** NSCLC cells were transfected with parkin siRNA and then stained with DAPI and TUNEL solutions. The total number of cells in a given area was determined using DAPI nuclear staining (fluorescent microscope). The green color in the fixed cells indicates TUNEL-labeled cells. **(E–F)** NSCLC cells were transfected with parkin siRNA, after which the expression of apoptotic proteins was examined by western blotting. β-actin protein was used as an internal control. Each band is representative of three independent experiments.

To investigate how parkin affects cancer cell growth, we transfected NSCLC cells with parkin siRNA using a transfection agent for 48 h, after which analysis of cell growth was performed using the MTT assay. Knockdown of parkin inhibited the growth of A549 and HCC-1588 lung cancer cells (Fig 5A and 5B, upper panels). Morphological observations indicated that upon parkin siRNA transfection, cells gradually decreased in size and attained a small, protruding shape (Fig 5A and 5B, lower panels). We also performed DAPI and TUNEL staining assays to determine whether the inhibition of cell growth by parkin siRNA was due to the induction of apoptotic cell death. Reversely, apoptotic cell death was significantly increased in parkin siRNA-treated A549 and HCC-1588 NSCLC cells (Fig 5C and 5D). Expression of cleaved caspase-3, an apoptotic regulatory protein, was also increased by knockdown of parkin (Fig 5E and 5F).

## Discussion

There has been a long-standing argument regarding the association between PD and cancer incidence. In this work, we studied how a PD-related gene, parkin, affects lung tumor growth. We confirmed a preferentially higher expression of parkin in NSCLC cells than in normal LL24 cells. In addition, the expression of parkin was found to progressively increase in human NSCLC tumor tissues (Grade I‒III) in a tumor grade-dependent manner. We also found that knockdown of parkin inhibited lung cancer cell growth and resulted in increased cell cycle arrest at the sub G0/G1 phase in NSCLC cells. We further found that parkin KO mice showed decreased lung tumor growth compared with WT mice. These data demonstrate that parkin KO inhibits lung tumor growth, suggesting that lung tumor development occurs less frequently in patients with PD.

p21 is a well-known inhibitor of cell cycle progression and can arrest cells in the G1/S and G2/M transitions by inhibiting the formation of a CDK2/cyclin E complex, which functions to restrict the G1/S phase of the cell cycle [32–33]. Notably, it has been reported that p21 can bind to CDK2, leading to the suppression of the CDK/cyclin complex. In this study, we showed that knockdown of parkin resulted in higher expression of phosphorylated p21 and its accumulation in the nucleus of NSCLC cells. Moreover, the p21/CDK2 complexes were found to be increased in parkin siRNA-treated lung cancer cells, and this increased association was correlated to the sub G0/G1 arrest in parkin siRNA-treated lung cancer cells. It has also been reported that the inactivation of p21 in normal human keratinocytes by HPV-16E7 oncoproteins results in higher CDK2/cyclin E activity, which favors DNA replication to occur [34]. It is also well established that p21 binds to PCNA and interferes with PCNA-dependent DNA polymerase activity, resulting in inhibition of DNA replication [28]. It was previously found that the interaction between PCNA and p21 induces G1 and G2 arrest in p53-deficient DLD1 human colon cancer cell lines [35]. DLD1 p21PCNA− human colon carcinoma cell lines expressing a mutant form of p21, which interfered with its interaction with PCNA, were not able to arrest at the G2/M checkpoint. Thus, it is possible that higher levels of p21 in parkin KO conditions result in an increased interaction with PCNA, thus inhibiting DNA synthesis and maintaining cells in a sub G0/G1-arrested state [36]. In this study, we found an increased interaction between p21 and PCNA or CDK2 after knockdown of parkin using siRNA. We also observed increased interactions between p21 and PCNA or CDK2 in urethane-induced lung tumors of parkin KO mice compared to those in WT mice. Furthermore, this increase in interaction continuously inhibited the expression of cell cycle proteins, such as CDK2 and cyclin E. Thus, these data indicate that maintaining p21 expression can result in increased interactions between p21 and PCNA or CDK2, resulting in cell cycle arrest, which ultimately inhibits lung tumor growth.

Our data indicate that higher p21 levels in parkin siRNA-treated cells and parkin KO mice are critical for cancer cell growth inhibition. However, it is unclear how parkin KO can restore p21 levels to control cell cycle arrest. We focused on the possibility that p21 acts a protein substrate of parkin through the ubiquitin-proteasome system (UPS), as parkin activates the UPS, a major protein degradation system [37]. We found an interaction between parkin and p21 by Octet and immunoprecipitation analyses. We have previously reported that the RING1 and RING2 domains of parkin are critical for its interaction with p21. Mutant parkin proteins (RING1 domain mutant, R275W and RING2 domain mutant, G430D) were observed to have reduced interactions with p21, as demonstrated by immunoprecipitation. We observed the ubiquitination and therefore degradation of p21 by cell free-in vitro and in vivo ubiquitination assays in mutant cells. p21 levels were significantly reduced in WT mice, but the expression of p21 was significantly recovered to steady-state levels in mutants with missense mutations (R275W and G430D). In addition, p21 degradation was reduced in parkin siRNA-treated cells. We also observed higher endogenous ubiquitination of p21 and lesser degradation of p21 in the lung tumor tissues of parkin KO mice than those in WT mice. p21/CDK2 and p21/PCNA complexes were also observed to a greater extent in parkin KO mice lung tumors. Generally, it has been reported that substrates bind to the RING domain of parkin where the ubiquitin is discharged and conjugated to the substrate [38]. Therefore, it is possible that parkin KO reduced p21 ubiquitination and degradation, leading to increased p21/CDK2 or p21/PCNA complex association, which ultimately resulted in the inhibition of tumor growth through cell cycle arrest.

Many epidemiological studies have indicated an association between the risk of cancer development and PD [4]. It has also been reported that PD gene family members (PARK1/4, PARK2, PARK5, PARK6, PARK7, PARK8, PARK9, and PARK15) are highly expressed in patients with NSCLC [6]. Therefore, our data indicate that parkin KO induced the repression of lung tumor development via increased p21/CDK2 or p21/PCNA complex association mediated cell cycle arrest due to failure of p21 degradation. Our study suggests that patients with PD are closely associated with low lung cancer incidence.

## Supporting information

S1 Fig Changes in cell cycle regulatory proteins after p21 knockdown in NSCLC cells. To identify how p21 controls cell growth through cyclin-dependent kinase complexes (CDKC), we transfected NSCLC cells with an siRNA against p21 for 48 h. Cell growth was then analyzed using the MTT assay. Knockdown of p21 inhibited the growth of A549 and HCC-1588 cells. We also observed that p21 knockdown increased the expression of parkin, CDK2, and cyclin E.
(TIF)
