# Supplementary figures and images for: Loss of parkin reduces lung tumor development by blocking p21 degradation
Source: PLoS One. 2019 May 21;14(5):e0217037. doi: 10.1371/journal.pone.0217037 (PMC6528990; doi:10.1371/journal.pone.0217037)

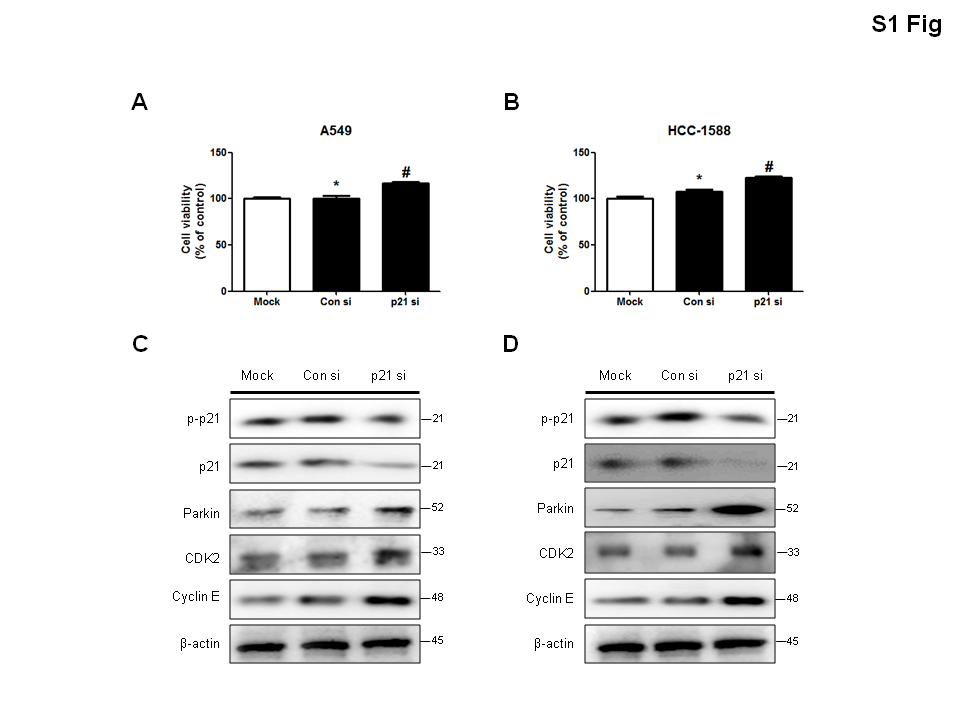

Supplement: S1 Fig — To identify how p21 controls cell growth through cyclin-dependent kinase complexes (CDKC), we transfected NSCLC cells with an siRNA against p21 for 48 h. Cell growth was then analyzed using the MTT assay. Knockdown of p21 inhibited the growth of A549 and HCC-1588 cells. We also observed that p21 knockdown increased the expression of parkin, CDK2, and cyclin E. (TIF) [file pone.0217037.s001.TIF]
